# Adaptation to Laterally Asymmetrical Visuomotor Delay Has an Effect on Action But Not on Perception

**DOI:** 10.3389/fnhum.2019.00312

**Published:** 2019-09-06

**Authors:** Chen Avraham, Mor Dominitz, Hana Khait, Guy Avraham, Ferdinando A. Mussa-Ivaldi, Ilana Nisky

**Affiliations:** ^1^Department of Biomedical Engineering, Ben-Gurion University of the Negev, Beersheba, Israel; ^2^Zlotowski Center for Neuroscience, Faculty of Health Sciences, Ben-Gurion University of the Negev, Beersheba, Israel; ^3^Department of Psychology, University of California, Berkeley, Berkeley, CA, United States; ^4^Helen Wills Neuroscience Institute, University of California, Berkeley, Berkeley, CA, United States; ^5^Department of Physiology, Feinberg School of Medicine, Northwestern University, Evanston, IL, United States; ^6^Department of Biomedical Engineering, McCormick School of Engineering and Applied Science, Northwestern University, Evanston, IL, United States; ^7^Shirley Ryan AbilityLab, Chicago, IL, United States

**Keywords:** visuomotor delay, reaching, line bisection, adaptation, transfer, visual perception, hemispatial neglect

## Abstract

When interacting with the environment, the sensorimotor system faces temporal and spatial discrepancies between sensory inputs, such as delay in sensory information transmission, and asymmetrical visual inputs across space. These discrepancies can affect motor control and the representation of space. We recently showed that adaptation to a laterally asymmetric delay in the visual feedback induces neglect-like effects in blind drawing movements, expressed by asymmetrical elongation of circles that are drawn in different workspaces and directions; this establishes a possible connection between delayed feedback and asymmetrical spatial processing in the control of action. In the current study, we investigate whether such adaptation also influences visual perception. In addition, we examined transfer to another motor task – a line bisection task that is commonly used to detect spatial disorders, and extend these results to examine the mapping of these neglect-like effects. We performed two sets of experiments in which participants executed lateral reaching movements, and were exposed to visual feedback delay only in the left workspace. We examined transfer of adaptation to a perceptual line bisection task – answers about the perceived midline of lines that were presented in different directions and workspaces, and to a blind motor line bisection task – reaching movements toward the centers of similar lines. We found that the adaptation to the asymmetrical delay transferred to the control of lateral movements, but did not affect the perceived location of the midlines. Our results clarify the effect of asymmetrical delayed visual feedback on perception and action, and provide potential insights on the link between visuomotor delay and neurological disorders such as the hemispatial neglect syndrome.

## Introduction

To perform accurate hand movements, the sensorimotor system gathers and integrates external information with internal predictions about the outcomes of action. During these processes, perception and action are modified to compensate for possible changes in the environment. Specifically, the sensorimotor system holds asymmetrical representation of spatial information in the hemispheres (Heilman and Valenstein, 1979; Ziemann and Hallett, 2001; Koch et al., 2011). Additionally, it also has to deal with time delays in sensory information transmission and delays between modalities (Miall et al., 1985; Miall and Jackson, 2006; Pressman et al., 2007; Di Luca et al., 2011; Nisky et al., 2011; Honda et al., 2012; Rohde et al., 2014; Avraham et al., 2017a; Farshchian et al., 2018). In this study, by investigating adaptation to laterally asymmetrical delay in the visual feedback, we set out to understand the processes of compensation for laterality and delay in perception and in the control of action.

To cope with time delays in the sensory feedback, our sensorimotor system relies on internal models. The internal models are representations of the motor apparatus and the environment that are used to predict the sensory consequences of motor commands, and thereby are allowing to cope with inherent feedback and processing time delays (Jordan and Rumelhart, 1992; Miall and Wolpert, 1996; Wolpert, 1997; Kawato, 1999). These models are updated when there are changes in our motor apparatus or in the environment. To evaluate updates in internal models, adaptation studies examined participants’ movements and space representation following exposure to visuomotor or force perturbations (Shadmehr and Mussa-Ivaldi, 1994; Cohn et al., 2000; Krakauer et al., 2000; Simani et al., 2007). During adaptation, the participants adjust to the new environment by modifying their movement kinematics and dynamics according to changes in the internal model. These modifications are demonstrated by the observation of aftereffects when the perturbation is removed (Shadmehr and Mussa-Ivaldi, 1994; Krakauer et al., 2000), and sometimes are also accompanied by perceptual biases (Colent et al., 2000; Goedert et al., 2010; Michel et al., 2018).

The nature of the changes in the internal model is investigated by examining generalization to movements performed in different spatial positions or limb postures (Krakauer et al., 2000; Donchin et al., 2003; Wang and Sainburg, 2005; Poh and Taylor, 2018), and transfer of adaptation to a different workspace (Shadmehr and Mussa-Ivaldi, 1994; Rotella et al., 2015) or to a different task (Shadmehr and Mussa-Ivaldi, 1994; Botzer and Karniel, 2013; Avraham et al., 2017a). When presented with a delay in visual feedback, participants initially overshoot the targets of reaching movements, but restore their original movement extent with adaptation, and exhibit aftereffects of undershooting the target (Botzer and Karniel, 2013; Avraham et al., 2018). Interestingly, the transfer of adaptation to delayed visual feedback causes elongation of blind reaching movements (Botzer and Karniel, 2013; Avraham et al., 2017a; Sulimani et al., 2017; Farshchian et al., 2018), and hence, visuomotor delays were proposed to be represented as a minifying visuomotor gain (Botzer and Karniel, 2013; Avraham et al., 2017a; Sulimani et al., 2017).

In our recent study (Avraham et al., 2018), we made the first steps toward linking between asymmetrical representation of spatial information in the hemispheres and adaptation to delayed visual feedback. We defined left and right workspaces as the left and right halves of the space in front of participants (with respect to the midline of their body), and studied adaptation to delayed visual feedback that was presented in either left, right, or both workspaces. We demonstrated a unique pattern of elongated transfer movements after adaptation to these asymmetrical delay conditions. However, because in that study all the movements were initiated in the center, movements in the left workspace were performed in leftward direction, and movements in the right workspace were performed in rightward direction. Therefore, we could not disassociate whether the representation depended on workspace or movement direction.

Previous studies also reported that motor adaptation affects perception. These studies showed evidence for the effect of kinematic (visuomotor rotation) and dynamic (force-field) perturbations on the perceived movement direction and location of the hand (Ostry et al., 2010; Mattar et al., 2012; Marius‘t Hart and Henriques, 2016). The effects were shown to be much smaller than the motor effects, but nevertheless robust and long-lasting (Cressman and Henriques, 2010; Ostry et al., 2010; Ruttle et al., 2016). In addition, perceptual training was also shown to improve motor learning (Darainy et al., 2013). However, in the case of adaptation to visuomotor delay perturbation, a recent study showed that after exposure to delayed visual feedback, the proprioceptive representation remains unaltered, as opposed to the control of action (Sulimani et al., 2017). In light of these contrasting results, an interesting open question is whether the unique visuomotor perturbation combining visuomotor delays and spatial laterality will result in perceptual effects.

One pathology that demonstrates a deficit in spatial and temporal processing of information for perception and action is *Hemi-spatial neglect* – a neurobehavioral deficit caused by brain damage. Neglect patients fail to perceive and respond to stimuli originating from their contralesional side, mostly their left side, consistently with right-brain damage. Neglect can involve a variety of impairments in spatial information processing for both perception and action, demonstrated in perceptual–attentional and motor-intentional spatial deficits (Bartolomeo et al., 1998; Adair and Barrett, 2008). In addition to the spatial deficits, some studies also reported temporal impairments, suggesting that neglect might be a spatial–temporal deficit (Robertson et al., 1998; Becchio and Bertone, 2006). Several clinical tests are used to diagnose spatial neglect (Adair and Barrett, 2008). Two prominent tests are the perceptual line bisection task and the motor line bisection task (Schenkenberg et al., 1980). The perceptual line bisection test uses a forced choice paradigm. A transected line is presented to participants who need to judge whether the transection mark is on the right side with respect to the midline. In the motor line bisection test, the participants are required to mark the center of a presented line. This means that the participants actually perform reaching movements toward the center of the lines that are presented to them.

In the current study, we adapted the perceptual and motor line bisection tests to investigate transfer of adaptation to asymmetrical delay in visual feedback that may cause transient neglect-like effects on perception and action. We aimed to extend our previous study by answering two questions. First, whether the asymmetrical elongation of movements following adaptation to laterally asymmetrical visuomotor delay will affect both perception and action. Second, whether the asymmetrical elongation is a result of representation of the laterally asymmetric perturbation with respect to the workspace in which the transfer movement was executed, or the direction to which it was oriented. We asked participants to perform lateral center-out reaching movements to both left and right targets, and presented them with visual feedback delay only in movements to the left targets. We tested transfer of adaption to both a blind motor line bisection task (*Action* group) and a perceptual line bisection task (*Perception* group). The blind line bisection movements were performed toward leftward or rightward directions in left or right workspaces. We found that adaptation to asymmetrical delay has an asymmetrical transfer to the motor task, but we found no evidence of transfer to the perceptual task. These results demonstrate a dissociation between the effects of adaptation on action and on perception. Overall, our results further establish the effect of lateral and temporal misalignment between modalities, and provide support for independent processing of sensory information in the motor and the perceptual systems.

## Materials and Methods

### Participants and Experimental Setup

**FIGURE 1:**
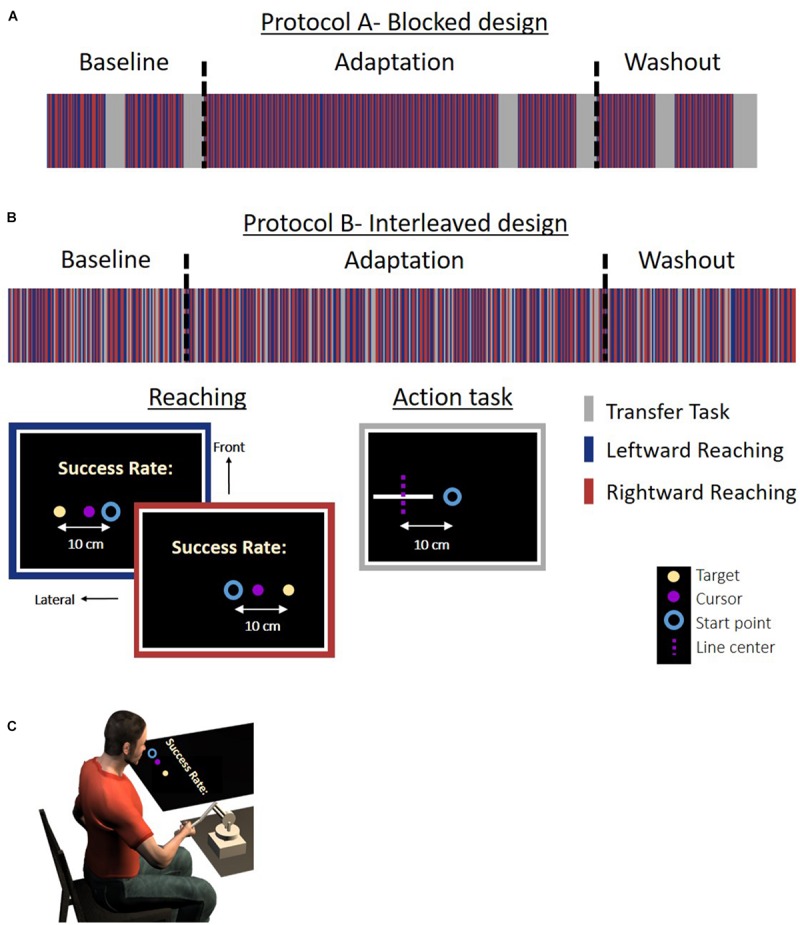
Experiment 1: protocol and setup for the *Action* group. **(A)** Experimental protocol for the *blocked* design. The experiment was divided to blocks of reaching movement and an action task. **(B)** Experimental protocol for the *interleaved* design. The reaching movements and task trials were randomly displayed throughout the experiment in a predetermined order. In the reaching trials, participants were required to move a cursor (magenta circle) between a start point (blue circle) and an end target (light yellow dot) to the left (blue frame) or the right (red frame) side of the task space. To motivate the participants, we presented a success rate representing the percentage of accurate trials (in which the participants hit the target) out of all reaching trials in the experiment until that time. In the action task (gray frame), the participants had to move their hand from the start position (blue circle) to the center of a white line without visual feedback. The dashed magenta line indicates the actual midline and was not presented to the participant. The line and start position were located at three different positions and were all aligned in the lateral axis. The experiment was divided into three sessions: Baseline, Adaptation, and Washout. During the Baseline and Washout sessions, the cursor movement in the reaching task was concurrent with the movement of the hand. During the Adaptation session, the visual feedback was delayed by 0.15 s in movements toward the left side. The stripes representing the different tasks are only for illustration, and the figure does not include the entire trials in the experiment. **(C)** Experimental setup. Participants held a haptic device, controlling a cursor displayed on a screen. The experiment was displayed on a screen that was located horizontally above participants’ hand (see the section “Materials and Methods” for more details).

**FIGURE 2:**
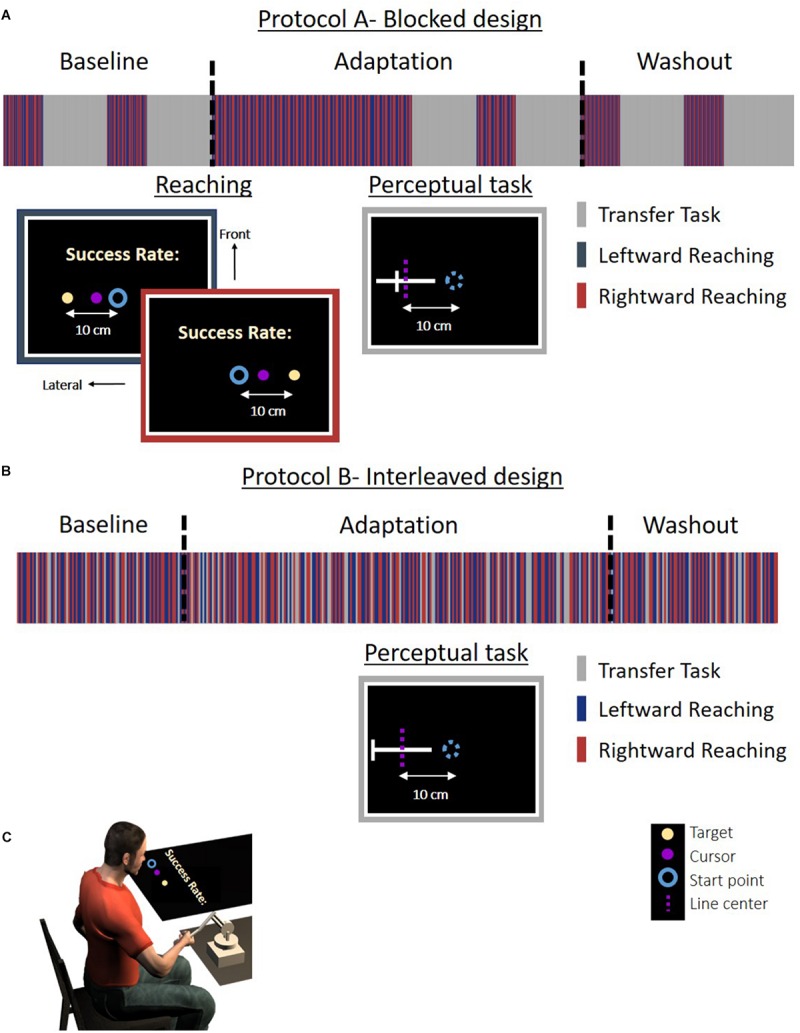
Experiment 1: protocol and setup for the *Perception* group. **(A)** Experimental protocol for the *blocked* design. The experiment was divided to blocks of reaching movement and a perceptual task. Reaching movements are the same as in Figure 1. In the perceptual task (gray frame), a white lateral line and probe (white frontal line) were presented. The participant was required to answer whether the probe is on the right side of the line. The dashed magenta line indicates the actual midline and was not presented to the participant. The lines were presented in the same locations as for the *Action* group. **(B)** Experimental protocol for the *interleaved* design. Reaching movements and task trials were randomly displayed in a predetermined order. Here, in the perceptual task, we displayed a white lateral line and a probe (white frontal line). The probe was located in the right or left edge of the line. Participants were required to move the probe to the midline by using the left and right arrows in the keyboard. The dashed magenta line indicates the actual midline and was not presented to the participant. The stripes representing the different tasks are only for illustration, and the figure does not include the entire trials in the experiment. **(C)** Experimental setup. The setup was similar to the *Action* group. Experiment 2 was similar to Experiment 1, except that here the lines were presented with a displacement of 5 cm along the frontal axis in both the action and the perception task.

Eighty-five right-handed healthy volunteers (ages 18–29 years, 40 females) participated in the study that was approved by the Human Subjects Research Committee of Ben-Gurion University of the Negev, Beersheba, Israel, after signing an informed consent form. The participants were all naive to the purpose of the experiment and were paid to participate. The experiment was administered in a virtual reality environment in which the subject held a PHANTOM^®^ DESKTOP^TM^ (Geomagic^®^) haptic device that was controlled by a custom-written C++ code. During the experiment, participants held the haptic device with their right hand, controlling a cursor that was displayed on a screen (Figures 1, 2). The cursor movement was synchronized with the hand movement, with a delay of 10 ms resulting from the control loop. The experiment was displayed on a screen located horizontally above the hand of the participants, and their upper body was covered by a sheet such that they could not see their hand. Hand movements were limited to the horizontal plane by an air sled wrist-supporter that reduced friction with the surface. The update rate of the control loop was 1000 Hz.

### Protocol

**FIGURE 3:**
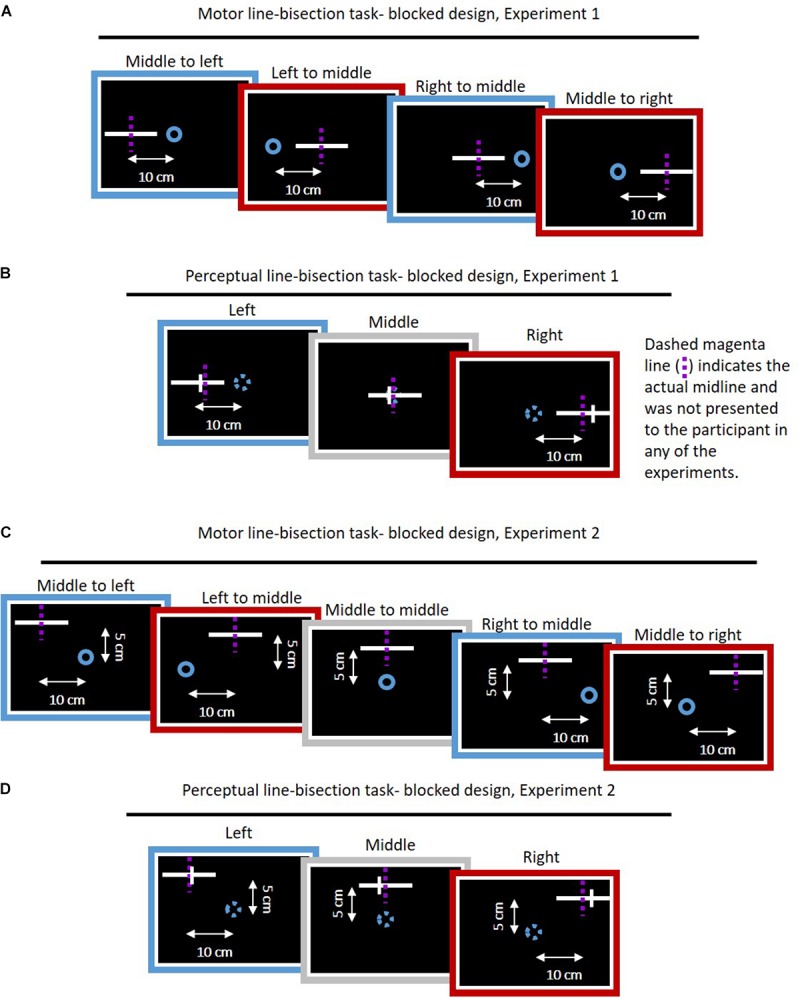
Task blocks of motor and perceptual line bisection task for the *blocked* design in both experiments. **(A)** Motor line bisection task for Experiment 1. On every trial, a start point (blue circle) and a straight line (white) were displayed, and participant were instructed to perform smooth movement toward the center of the line. The dashed magenta lines indicate the actual midline and were not presented to the participant. Every task block consisted of four different movement types: left to middle, middle to left, middle to right, and right to middle. For every movement, we had five repetitions, such that in each block the participants performed 20 movements. **(B)** Perceptual line bisection for the *blocked* design in Experiment 1. In this task, we presented a straight lateral line (white) in three different locations of: left, middle, and right. The line was bisected with a frontal line (white small line), and the participant was required to answer whether the frontal line is on the right side with respect to midline. The start point omitted in the perceptual task. We had eight frontal lines on different locations on the lateral line and each frontal line was displayed four times, such that we had 96 trials in every block. **(C)** Same as **(A)**, for Experiment 2. Here the lines were 5 cm displaced along the frontal axis. Furthermore, we had additional movement of middle to middle (gray), such that every block contained 25 movements. **(D)** Perceptual line bisection for Experiment 2. Same as **(B)**, except here the lines were also 5 cm displaced along the frontal axis. Action task trials of the *interleaved* design were identical to the *blocked* design. Perceptual task trials of the *interleaved* design were similar to the ones presented here, except the frontal line was located only on the left or right edge of the presented line.

We conducted two experiments. In each experiment, we had two different protocols and two groups for each protocol (overall seven groups with *N* = 10 in each group, and one group with *N* = 15). In all the experiments, the participants were asked to perform reaching movements to left or right targets relative to a central start position (Figures 1, 2). To assess the effect of asymmetrical temporal perturbation, we applied a delay of 0.15 s only in the left workspace. We probe for the effect of the delay on action and perception with a transfer task that was applied in designated blocks throughout the experiment (protocol A – *blocked* design, Figures 1A, 2A) or in random trials throughout the experiment (protocol B – *interleaved* design, Figures 1B, 2B). The transfer task was either a motor line bisection task without visual feedback (*Action* group, Figure 1), or a perceptual line bisection (*Perception* group, Figure 2). In Experiment 1, the lines that were presented during the task were aligned with the start position along the lateral dimension (Figures 3A,B). In Experiment 2, the lines were 5 cm away from the start position in the frontal axis (Figures 3C,D). The trials were presented in a random and predetermined order.

#### Experiment 1

In the protocol of the reaching task, there was no difference between the *blocked* and *interleaved* protocols. A trial was initiated when participants placed a circular white cursor, 1 cm diameter, inside a starting point, a blue hollow 2 cm diameter circle, which was placed in the middle of the screen. The participant performed a smooth point-to-point reaching movement by moving the cursor from the starting point to a circular yellow target, 1 cm diameter, which appeared in the left or the right side of the workspace, at 10 cm away from the starting position. In each reaching block, the trials order was random and predetermined between left and right targets. Movement started from rest at the start position for 1 s, with a color-cue of the cursor, and ended when the velocity of the haptic device was <0.01 m/s. At the end of the trial, the visual cursor was omitted and the hand of the participant was returned passively to the start position by a spring-like force that was applied by the haptic device. Following the movement, during the passive return to the start position, we presented a feedback based on the accuracy and the velocity of the movement. We defined accurate movements as those that ended within the target, with a velocity that ranged between 0.3 and 0.5 m/s. When the maximum velocity was <0.3 m/s, the word “Faster” appeared on the screen, and when the velocity was >0.5 m/s, the word “Slower” was displayed. Moreover, the position of the cursor at the end of the movement was displayed for 1.5 s, with a color cue that indicated the accuracy of the movement (green for accurate movement and red for inaccurate movement). We also presented a success rate corresponding to the percentage of successful trials from all reaching trials in the experiment until that time.

In the *blocked* design, the participants performed a transfer task in several blocks throughout the experiment (two blocks in each of the baseline, adaptation, and washout sessions). The *Action* group (*N* = 10) performed a blind motor line bisection task. In the blind motor line bisection task, participants performed reaching movements from the same starting hollow blue circle toward the middle of a 10-cm line without visual feedback of their cursor. There were three possible locations for the lines and three start points (left, right, and middle), which were all laterally aligned (Figure 3A). Accordingly, we had four movement types of leftward and rightward lateral movements in each (left and right) workspace. To initiate a movement, the participant placed the cursor into the starting point, after which the cursor disappeared. Similarly to the lateral reaching movements, movement ended when the velocity was <0.01 m/s, and the haptic device applied a spring-like force that returned the hand to the next start position.

The *Perception* group (*N* = 10) performed a perceptual line bisection task. In this task, the participants were presented with a 10-cm line located in the same positions as in the action task, with a probe (frontal small line) of 1 cm length that was positioned in one of eight different locations on the line (Figure 3B). The probes were 1 mm apart, while the most distant were located 3.5 mm from the center of the line, symmetrically. This small increment of 1 mm was chosen empirically to observe whether a perceptual bias existed even at baseline. In each trial there were only one line and one probe. The participants were asked to answer whether the probe was located rightward relatively to the center of the line; that is, a yes or no response. In this task, we did not present the start point, and participants were instructed to remain on the start position.

In the *interleaved* design, the task trials were randomly intertwined throughout the experiment (Figures 1B, 2B). The *Action* group (*N* = 15) performed the same task as in the *blocked* design (Figure 3A). However, the *Perception* group (*N* = 10) performed a modified perceptual line bisection task. In this task, we presented a 10-cm line in the same locations as in the *blocked* design, and a 1-cm probe that was located in the right or the left edge of the presented line (similar to Figure 3B, except from the location of the probe that was in the right or left edge). The participants were required to move the probe to the perceived midline by pressing the right and left arrows in the keyboard without any restrictions on the amount of arrow pressing. When participants decided that the probe is indeed in the midline, they were required to press the up or down arrows, in order to move to the next trial.

We chose to implement two different protocols to probe the effect of laterally asymmetrical delay on perception and action. The *blocked* design was used to allow fitting the psychometric functions for the perceptual task’s results on data from the same phase in the experiment. The *interleaved* design allowed a more sensitive examination for transfer of adaptation, likely because it was more resistant to a possible accumulation of decay effects than the *blocked* design.

The experiment was divided into three sessions: Baseline, Adaptation, and Washout (Figures 1, 2). In the *blocked* design, each session consisted of two blocks of reaching movements and two task blocks. In the Baseline and Washout sessions, the reaching block contained 60 movements, and in the Adaptation session the first reaching block contained 360 movements and the second contained 60 movements. For the *Action* group, the task blocks consisted of 20 trials, such that we had five repeats for each movement type. For the *Perception* group, each task block consisted of 96 trials (four repeats for each line in every block – overall eight repeats for each line in every session). The purpose of having two blocks of reaching in every session was to reinforce the learning before the task block, such that during the task trials the adaptation process will not completely vanish. In the *interleaved* design, we had 160 reaching movements in the Baseline and Washout sessions, and 416 reaching movements in the Adaptation session. For the *Action* (*Perception*) group, we presented 40 (36) task trials in the Baseline and Washout sessions, and 104 (102) task trials in the Adaptation session.

During the Adaptation session of both protocols, the visual feedback in the reaching task was delayed by 0.15 s for leftward movements. The leftward movements were also performed in the left workspace. At the task trials, no perturbation was applied and there was no visual feedback of the movement of the hand. The entire experiment lasted approximately 90 min with four breaks of 2 min every 120 or 160 reaching trials for the *blocked* and *interleaved* design, respectively.

#### Experiment 2

To test the width of the generalization of the adapted representation, we chose to test the transfer of adaptation to movements that have a forward component, and performed a second experiment. The experimental setup was identical to Experiment 1. However, here the lines in the transfer trials were located 5 cm away from the starting point in the frontal axis, such that we had five types of movement in the action task: leftward and rightward diagonal movements in each workspace (angle of 26.57° and 116.57° for the left and right lines, respectively. The angle is calculated with respect to the positive lateral axis) and one frontal movement. Therefore, in the *blocked* design every task block consisted of 25 trials, and in the *interleaved* design the Adaptation session consisted of 105 task trials. The number of trials and order of the perceptual task was similar in both experiments.

### Data Analysis

Throughout the experiment, we recorded position and velocity at 200 Hz (we down sampled the data from the experiment). The results were analyzed off-line using custom-written MATLAB^®^ code (The MathWorks, Inc., Natick, MA, United States). In the reaching movements with visual feedback, we examined the amplitude of the movements. The amplitude was calculated as the maximum distance along the lateral axis. In the action task, we examined the lateral deviation of participants’ end point position from the center of the presented line.

In the perception task of the *blocked* design, we evaluated the perceived midline location from the response to each presented probe. First, we computed the probability for a positive response that indicates the probability for the participant to perceive the probe on the right side with respect to midline. Then, we fitted a psychometric curve to the calculated probability using MATLAB toolbox psiginfit(). Finally, we calculated the Point of Subjective Equality (PSE) that corresponds to the location of the probe where the probability for positive answer is 0.5, that is, the perceived location of the midline.

In the perception task of the *interleaved* design, we evaluated the changes in the perceived midline by examining the distance between the final location of the probe to the actual midline.

### Statistical Analysis

In the reaching task, the effect of the laterally asymmetric delay was assessed by comparing the changes in the amplitude of the movements in each group between the different stages of the experiment: Late Baseline (LB), Early Adaptation (EA), Late Adaptation (LA), and Early Washout (EW). The movements that were taken into consideration were five first movements in Adaptation and Washout (EA and EW, respectively), and the five last movements in Baseline and Adaptation (LB and LA, respectively). For both groups, we used three-way repeated measures ANOVA with the amplitude of movements as the dependent variable, and with the following independent factors: one between participants factor of Group (*Action*/*Perception*), and two within-participants factors of Direction (Leftward/Rightward) and Stage (LB/EA/LA/EW), including interactions.

In the action task, we used one-way repeated measures ANOVA for each movement type. We compared the two task blocks in the Adaptation session (LA #1 and LA #2) and the first task block in the Washout session (EW) in the *blocked* design, and LA and EW in the *interleaved* design, all relatively to the end of the Baseline.

Then, to answer the question whether the mapping following adaptation to laterally asymmetric delay depends on the direction or the workspace of movement, only for Experiment 1, we analyzed the results according to direction and workspace separately. We used two-way ANOVA with two within factors of Stage (LB/LA1/LA2/EW for the *blocked* design and LB/LA/EW for the *interleaved* design), Direction or Workspace (Left/Right) and the interaction between them. In the perception task, we used one-way repeated measures ANOVA model. In the *blocked* design, the dependent variable was the PSE values for every line, and the independent factor was the stage of the experiment (Baseline/Adaptation/Washout). The PSE from both blocks in the Adaptation and the Washout sessions was compared to the PSE from the two blocks in the Baseline session. In the *interleaved* design, we compared the deviation from midline in the end of the Adaptation session (LA) and in the beginning of the washout (EW), to the end of the Baseline, by using one-way repeated measures ANOVA model.

Significant effects were defined as those at the *p* < 0.05 probability level. When significant main or interaction effects were found, *post hoc* testing with Holm’s correction was conducted to identify the source of the differences. To examine whether the number of participants is sufficient for this analysis, we calculated the power of the ANOVA test with a parametric bootstrap test. We repeatedly generated random samples from a normal distribution, and calculated the percentage of statistically significant effects. The parameters of the normal distribution were calculated from the data. Based on examination of the data, the desired effect size (the mean of the normal distribution) was set to 1.5 cm, and the variance was determined based on the calculated variance of each group. The number of participants was chosen as the one that resulted in power >0.75. For the perceptual effects, we also calculated the power of the ANOVA test with a parametric bootstrap test. Here, because effects on perception are typically smaller than the effects on action (Ostry et al., 2010) an acceptable size of an effect was determined as 10% from the effect on action, and the variance was calculated from the data.

## Results

### Reaching Movements

**FIGURE 4:**
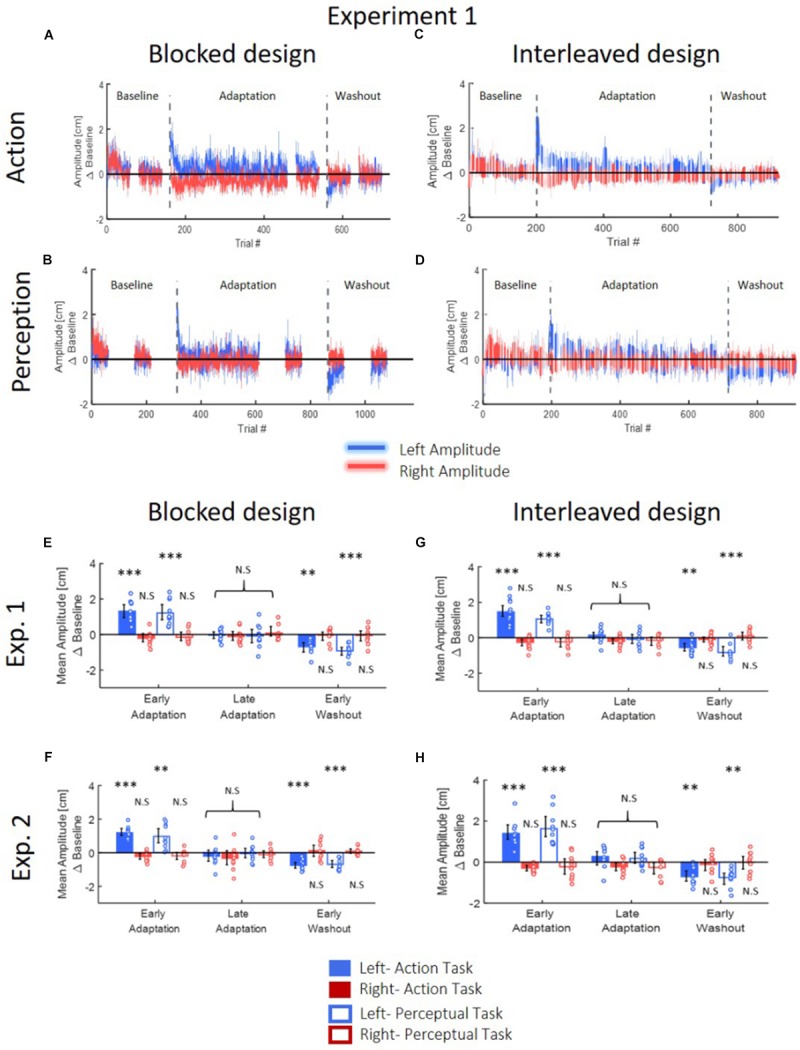
Reaching movements for Experiment 1 **(A–D)**. The results of Experiment 2 are similar. **(A)** Amplitude (line) and 95% confidence intervals (shaded region) of the leftward and rightward movements for the *Action* group of the *blocked* design. Results are presented after subtraction of the movement amplitude at the end of the baseline session. Positive (negative) value indicates overshoot (undershoot) in the direction of movement. The leftward movements show typical pattern of adaptation: overshoot when the perturbation is applied and undershoot when the perturbation is unexpectedly removed. The rightward movements are unaffected from the asymmetrical delay. **(B)** Same as **(A)** for the *Perception* group. **(C,D)** Same as **(A,B)** for the *interleaved* design. Here, task trials (white lines) were interleaved throughout the experiment. **(E)** Mean amplitude of the first and last five movements of the adaptation stage and the first five movements of the washout, compared to the last five movements of the baseline, for both *Action* and *Perception* groups of the *blocked* design in Experiment 1. Colored circles represent the mean amplitude of each subject, and error bars represent 95% confidence interval. ^∗∗^*p* < 0.01, ^∗∗∗^*p* < 0.001. No difference is observed between the two groups. **(F)** Similar to **(E)** for the *interleaved* design in Experiment 1. **(G,H)** Similar to **(E,F)** for Experiment 2. The observed results of the reaching movements are similar between the two groups and two experiments.

To assess the adaptation, we examined the change in the amplitude of the lateral reaching movements. This analysis was done to assure that the participants of all groups in all the experiments have adapted to the asymmetrical delay in the visual feedback by selectively modifying their reaching movements in the left workspace. Our results showed that for all groups, participants adapted and modified their movements when they were exposed to delay that was introduced exclusively in leftward movements in the left workspace (in Figures 4A–D, the results of the adaptation curves are displayed only for the Experiment 1, but the results of Experiment 2 are very similar). When the perturbation was first applied, participants over-reached the target only in movements toward the left target. After further exposure to the perturbation, participants returned to baseline performance. Initially, soon after the beginning of the exposure and as participants started adapting their leftward movements, there was also a small change in the rightward movements. This result was also observed in our previous study (Avraham et al., 2018) and it might stemmed from the fact that initially the participants interpret the perturbation as spatial shift. However, this change quickly disappeared as participants built a representation of the laterally asymmetric perturbation, and it was not statistically significant in the overall analysis. After the delay was unexpectedly removed, the leftward movements demonstrated an aftereffect of under-reaching, and as expected, we saw no aftereffects on rightward movements.

These observations were supported by our statistical analysis that is summarized in Table 1 and in Figures 4E–H. We divided the experiment to four stages of LB (five last movement before exposure to delay), EA (five first movements with the presence of delay), LA (five last movements with the presence of delay), and EW (five first movements after removing the delay). For both groups, we performed a three-way repeated measures ANOVA with the amplitude of movements as the dependent variable, and with the following independent factors: one between participants factor of Group (*Action/Perception*), and two within-participants factors of Direction (Leftward/Rightward) and Stage (LB/EA/LA/EW), including interactions. The statistical analysis yielded a significant interaction between movement direction and stage, and therefore, we conducted a *post hoc* paired *t*-test. In both experiments, protocols and groups, we found a typical pattern of adaptation in leftward movements: a significant over-reach in the EA stage, no difference in LA stage, and undershoot in EW stage. In contrast to leftward movements, where the perturbation was applied, throughout the experiment there was no change in rightward movements (Figures 4E–H). *These results indicate that participants were able to adapt to the asymmetrical visuomotor delay by asymmetrically modifying their motor commands*.

**TABLE 1:** Statistical analysis for the reaching movements in both experiments.

		**Experiment 1**				**Experiment 2**			
		***Blocked* design**		***Interleaved* design**		***Blocked* design**		***Interleaved* design**	
		**Action group**	**Perception group**	**Action group**	**Perception group**	**Action group**	**Perception group**	**Action group**	**Perception group**
Groups	*F*	*F*_1__,__18_ = 0.35		*F*_1__,__23_ = 2.09		*F*_1__,__18_ = 0.002		*F*_1__,__18_ = 0.31	
	*p*	0.56		0.16		0.96		0.58	
	η^2^	0.003		0.004		0.01		0.001	
Movement direction	*F*	*F*_1,18_ = 0.46		***F*_1,23_ = 93.51**		*F*_1,18_ = 1.24		***F*_1,18_ = 44.84**	
	*p*	0.51		**1.43*e*−9**		0.28		**2.79*e*−6**	
	η^2^	0.001		**0.06**		0.002		**0.04**	
Stage	*F*	***F*_3,54_ = 46.95**		***F*_3,63_ = 55.33**		***F*_3,54_ = 25.74**		***F*_3,54_ = 43.43**	
	*p*	**4.5*e*−15**		**2.46*e*−18**		**1.78*e*−10**		**2.03*e*−14**	
	η^2^	**0.25**		**0.25**		**0.22**		**0.26**	
Direction and stage	*F*	***F*_3,54_ = 63.2**		***F*_3,63_ = 114.63**		***F*_3,54_ = 96.92**		***F*_3,54_ = 63.8**	
	*p*	**1.14*e*−17**		**9.53*e*−27**		**9.99*e*−22**		**9.32*e*−18**	
	η^2^	**0.33**		**0.42**		**0.4**		**0.39**	

*Post hoc t*-test for leftward movements in the different stages compared to the end of Baseline									

EA stage	*d*	**2.95**	**2.02**	**3.11**	**3.81**	**3.11**	**1.99**	**3.07**	**2.57**
	*t*	***t*_18_ = 6.29**	***t*_18_ = 5.84**	***t*_23_ = 10.6**	***t*_23_** = **6.29**	***t*_18_ = 6.55**	***t*_18_ = 5.36**	***t*_18_ = 6.05**	***t*_18_ = 1.72**
	*p*	**0.0002**	**0.0004**	**6.2*e*−9**	**5.6*e*−5**	**0.0001**	**0.001**	**0.0002**	**3.82*e*−5**
LA stage	*d*	−0.07	−0.16	0.4	−0.30	−0.45	−0.17	0.63	0.54
	*t*	*t*_18_ = 0.19	*t*_18_ = 0.53	*t*_23_ = 1.41	*t*_23_ = 0.71	*t*_18_ = 1.21	*t*_18_ = 0.31	*t*_18_ = 1.72	*t*_18_ = 1.13
	*p*	1	1	1	1	1	1	1	1
EW stage	*d*	**−1.48**	**−1.73**	**−1.63**	**−2.51**	**−2.68**	**−2.64**	**−2.42**	**−2.49**
	*t*	***t*_18_ = 5.42**	***t*_18_ = 7.41**	***t*_23_ = 4.96**	***t*_23_ = 6.04**	***t*_18_ = 5.36**	***t*_18_ = 5.36**	***t*_18_ = 5.41**	***t*_18_ = 5.40**
	*p*	**0.001**	**1.9*e*−5**	**0.001**	**0.0001**	**1.9*e*−5**	**6.5*e*−5**	**0.002**	**0.001**

*The results show a significant difference (bold) between leftward movements performed in the different stages.*

### Action Task – Line Bisection

Next, we aimed to answer two questions about the *transfer of adaptation*: (1) whether the selective adaptation to an asymmetrical delay in the visual feedback transferred to the blind line bisection movements and (2) whether the transfer depends on the workspace in which the movement is performed or on the direction of the movement. To assess the transfer of adaptation, we analyzed the distance from the end point location of participants’ movement to the actual center of the line in the lateral axis.

**FIGURE 5:**
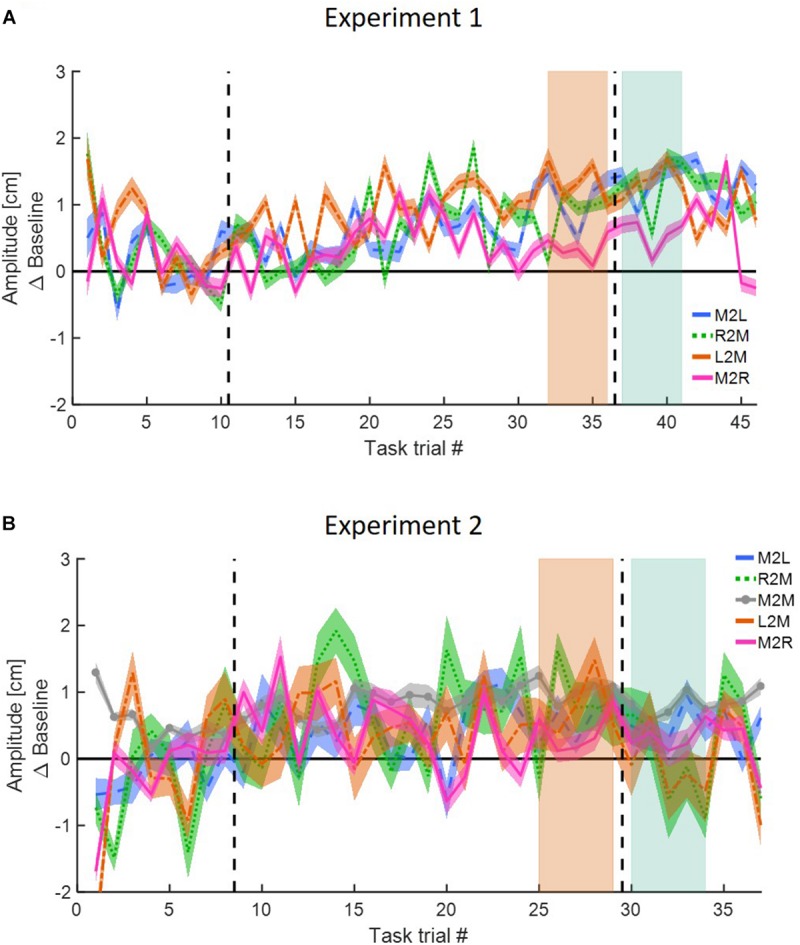
Movement amplitude in the lateral axis for the *interleaved* action task in both experiments. **(A)** Experiment 1. Movement amplitude for the different directions and locations. Results are presented for each line separately: leftward movement in the left workspace (M2L, blue), leftward movement in the right workspace (R2M, green), rightward movement in the left workspace (L2M, orange), and rightward movement in the right workspace (M2R, pink). Shaded regions represent the five last movements in the end of adaptation session (light orange) and five first movements at the beginning of the washout (light green). The results show that for all movement types there is an increase in the amplitude at the beginning of the adaptation (until roughly task trial 25), but only for rightward movements at the right workspace the amplitude decrease before the end of adaptation. **(B)** Same as **(A)** for Experiment 2. The results show no consistent increase or decrease in the amplitude during the entire experiment.

In the *interleaved* design, we first qualitatively examined the time course of the target overshoot in all task trials throughout the experiments compared to the end of the baseline task trials (Figure 5). In Experiment 1 (Figure 5A), we saw an increase in the amplitude during the adaptation in all movement types until roughly the middle of the adaptation phase (around task trial 25). Interestingly, this effect persisted and continued to increase until the LA and EW stages for all movement types except for rightward movements in the right workspace (M2R, pink line). This suggests that the asymmetrical delay transferred to blind lateral leftward line bisection movements in the left workspace and was generalized to leftward movements in the right workspace and rightward movements in the left workspace. In contrast, in Experiment 2 the variability was much larger, and there was no consistent effect on the calculated distance between the participants (Figure 5B).

To support these qualitative observations with statistical analysis, we divided the data to different stages and examined the effect of the applied perturbation on the change in the amplitude between the different stages. This analysis was performed in both the *blocked* and the *interleaved* design. In the *blocked* design, we examined the changes in the calculated distance between the two task blocks in Adaptation (all five movements from each task block – LA #1 and LA #2) and during the Washout (all five movements from the first task block in Washout – EW) relatively to the end of the Baseline (all five movements from the last task block in Baseline – LB) session. We looked at the two task blocks of adaptation separately to examine how the effect on transfer movements developed throughout adaptation. In the *interleaved* design, we probe for the changes in movements’ amplitude during the end of the Adaptation (five last task trials in Adaptation – LA) and beginning of the Washout (five first task trials in Washout –EW) sessions relatively to the end of the Baseline (five last task trials in Baseline – LB) session. In our analysis of the washout session, we found no difference between the beginning and end of this session in both protocols. Therefore, to remain consistent with our previous study (Avraham et al., 2018), and to focus on our original research questions on short-term delay effects on generalization across direction and workspace and on perception, we decided to include in our analyses only the early stage of Washout.

**FIGURE 6:**
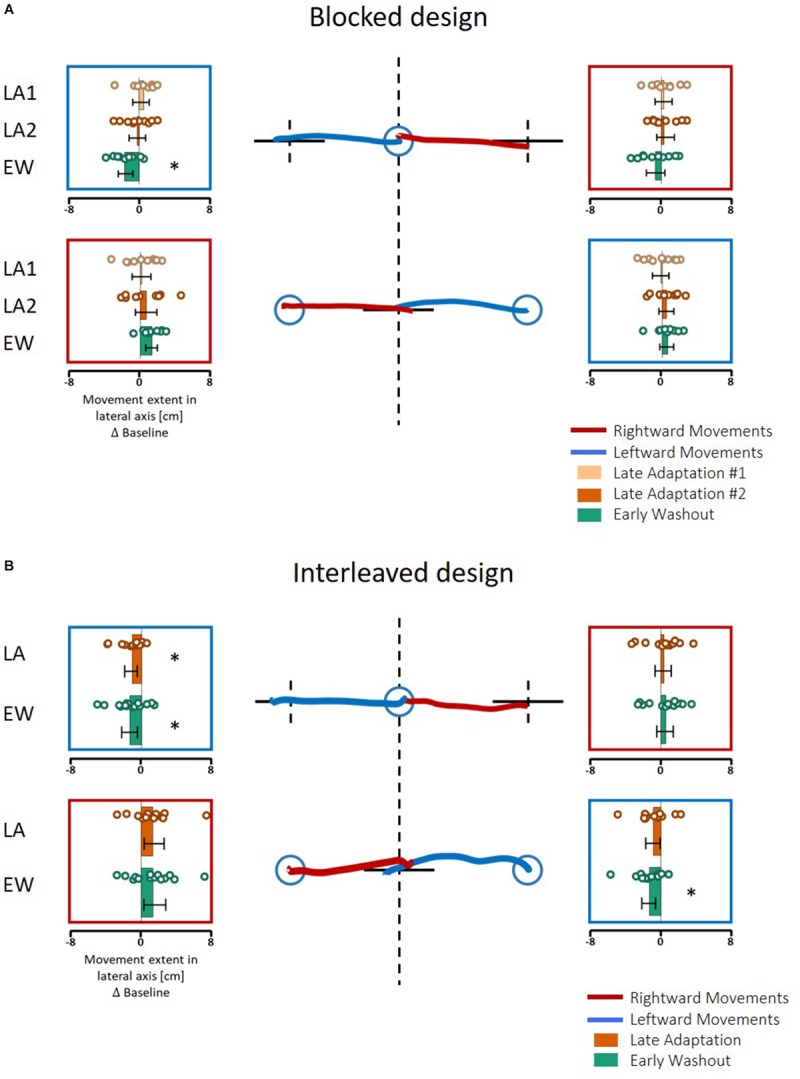
Spatial deviation from the center of the presented line along the lateral axis in the action task of Experiment 1. **(A)** *Blocked* design. At the center, examples of individual movements of a typical subject from the start point (blue circle) toward the center of the presented line (solid black line) in the left (blue) or right (red) directions. Dashed black lines show the actual center of the line and were not presented during the experiment. Panels around the center present the mean deviation in the stages of Late Adaptation 1 (light orange), Late Adaptation 2 (dark orange), and Early Washout (green) compared to the Late Baseline (LB). Colored circles represent the spatial deviation of each subject, and error bars represent 95% confidence interval. ^∗^*p* < 0.05. The panels are located spatially to represent the location and direction of the movement. The results suggest an elongation of leftwards movements performed in the left hemispace. **(B)** Similar to **(A)** for the *interleaved* design. Here, we analyzed the five last movements in Adaptation (Late Adaptation, dark orange) and five first movements in Washout (Early Washout, green) compared to the five last movements in the Baseline. The results suggest an elongation of leftward movements performed in both workspaces.

First, we examined the effect of the adaptation on each line separately by performing one-way repeated measures ANOVA for each movement type (the results of the entire analysis are summarized in Table 2, statistically significant effects are marked in Figure 6). In Experiment 1, we saw inconsistent results between the two protocols. In the *blocked* design, only in the Washout session, the deviation of leftward movements in the left workspace increased (*d* = −0.89, *t*_9_ = 3.4, *p* = 0.02, Figure 6A). The deviation of rightward movements in the left workspace also increased during Washout, but it was not statistically significant (*d* = 0.57, *t*_9_ = 2.46, *p* = 0.11). The results of the *interleaved* design showed a more robust effect of the transfer of adaptation, demonstrated in a deviation of leftward movements in the left workspace observed not only in the beginning of the Washout stage, but also in the end of the Adaptation session (LA: *d* = −0.67, *t*_14_ = 2.42, *p* = 0.02, EW: *d* = −0.74, *t*_14_ = 3.05, *p* = 0.017). Interestingly, with this more sensitive design, at the washout stage we also found that the transfer effects of adaptation generalized to leftward movements that were performed in the right workspace (LA: *d* = −0.58, *t*_14_ = 1.89, *p* = 0.07, EW: *d* = −0.84, *t*_14_ = 2.89, *p* = 0.02). In addition, we also found a significant main effect of stage in the analysis of rightward movements that were performed in the left workspace (η^2^ = 0.23, *F*_2,28_ = 4.26, *p* = 0.02), but even though the sizes of the effects were large (LA: *d* = 0.82, *t*_14_ = 2.22, *p* = 0.086, EW: *d* = 0.78, *t*_14_ = 2.08, *p* = 0.112, Figure 6B), the *post hoc t*-test with the multiple comparison correction did not yield statistically significant effect. In contrast, the transfer effects to rightward movements in the right workspace were much smaller and not statistically significant (LA: *d* = 0.22, *t*_14_ = 0.68, *p* = 1, EW: *d* = 0.35, *t*_14_ = 1.11, *p* = 0.57, Figure 6B). To conclude, *the only movements that were clearly not affected by the adaptation to asymmetrical delay in the visual feedback in none of the stages were rightward movements in the right workspace*.

**TABLE 2:** Statistical analysis for the motor line bisection task for each of the different movements in the two experiments.

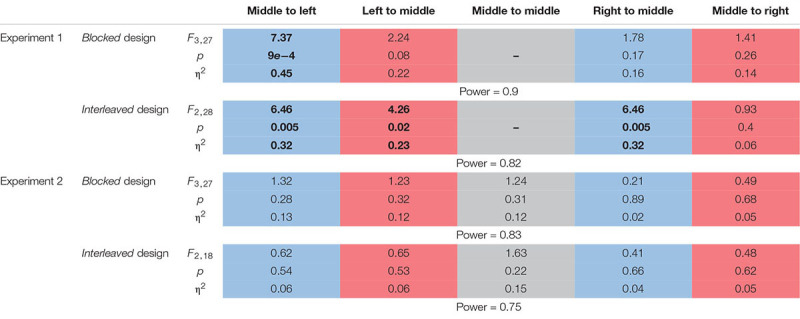

*We observed significant effects (bold) only for lateral leftward movements in the left workspace in the *blocked* design, and for lateral leftward movements performed in the right workspace in the *interleaved* design. The blue, red, and grey colors indicate leftward, rightward, and forward movements.*

**FIGURE 7:**
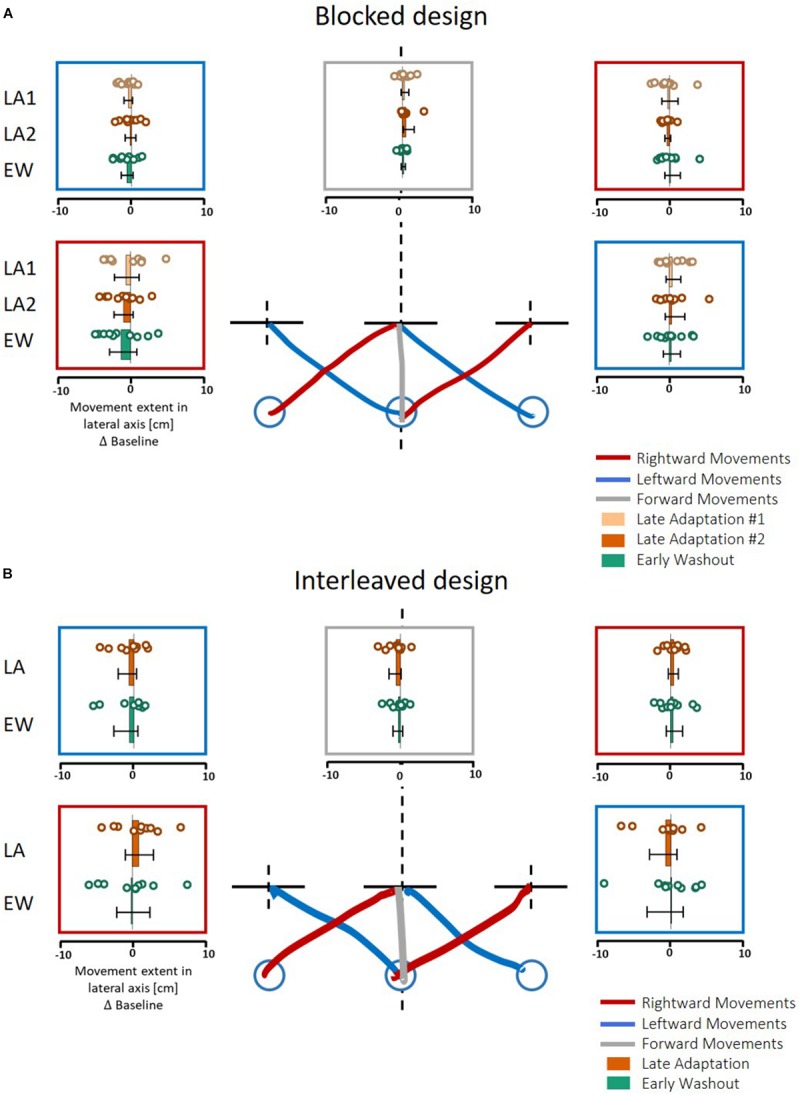
Results of action task in Experiment 2. **(A)** *Blocked* design. Results are presented in a similar manner as in Figure 6A, except here participants also did forward (gray) and diagonal movements. **(B)** Similar to **(A)** for the *interleaved* design. The analysis was performed on the five last movements in Adaptation (Late Adaptation, dark orange) and five first movements in Washout (Early Washout, green) compared to the five last movements in the Baseline. The results suggest no spatial deviation of bisection movements.

In contrast to these results, in both protocols of Experiment 2, we found no transfer effects in the motor line bisection task; i.e., participants’ movement toward the center of the line showed no deviation from the actual center (Figure 7, statistical analysis is summarized in Table 2). These results suggest that *the transferred effect is specific to purely lateral movements* and is not evident for movements that include a sagittal component (either diagonal or purely forward movements).

**FIGURE 8:**
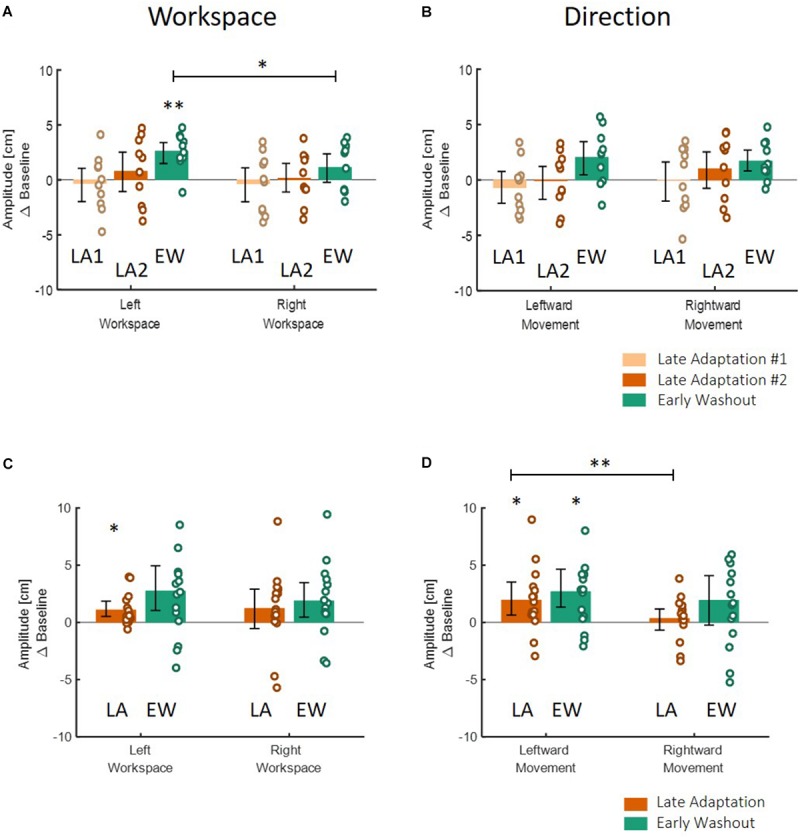
Spatial deviation from the center of the presented line along the lateral axis in the action task according to workspace **(A,C)** and direction **(B,D)** for the *blocked* design **(A,B)** and the *interleaved* design **(C,D)** in Experiment 1. **(A)** Deviation of movements according to the spatial location in which they were performed, in the different stages of Late Adaptation 1 (light orange), Late Adaptation 2 (dark orange), and Early Washout (green). Colored circles represent the calculated spatial deviation of each subject, and error bars represent 95% confidence interval. ^∗^*p* < 0.05, ^∗∗^*p* < 0.01. The results show a significant spatial elongation of movements performed in the left workspace during EW stage. This deviation is also different from right-workspace deviation. **(B)** Deviation of movements according to the direction toward which they were performed. Bars and colors are as in **(A)**. Results show no direction-related effects. Panels **(C,D)** are as **(A,B)** for the *interleaved* design. Here, we analyzed the different stages of Late Adaptation (dark orange) and Early Washout (green). The results suggest no significant effect of direction or workspace.

To answer the second question about the transfer of adaptation, we grouped the movements according to the direction or the workspace in which they were performed, and calculated the mean amplitude of the movement. We performed two-way repeated measures ANOVA with two within factors of Stage (LB/LA1/LA2/EW for the *blocked* design and LB/LA/EW for the *interleaved* design) and Direction or Workspace (Left/Right) including the interaction between them. The results of this analysis are shown in Figures 8A,B (left panel for the grouping according to workspace – left and right, and right panel for grouping according to movement direction – leftward and rightward). Statistical results are summarized in Table 3. In the *blocked* design, the results of workspace analysis showed statistically significant interaction between stage and workspace (η^2^ = 0.03, *F*_3,27_ = 3.4, *p* = 0.03). We found that a significant elongation of movements was exhibited only in the left workspace, and only during the Washout stage (*d* = 0.75, *t*_9_ = 4.99, *p* = 0.0045). In addition, we also found a significant difference between the amplitude in the right and left workspace observed in the EW stage (*d* = 0.76, *t*_9_ = 2.38, *p* = 0.04, Figure 8A). No similar pattern was observed in the direction analysis (Figure 8B).

**TABLE 3:** Results of the workspace-and-direction analysis for the results of Experiment 1.

		**Workspace**			**Direction**		
		**η^2^**	***F***	***p***	**η^2^**	***F***	***p***
*Blocked* design	Stage	**0.23**	***F*_3,27_ = 3.92**	**0.01**	**0.22**	***F*_3,27_ = 3.92**	**0.01**
	Side	0.02	*F*_1,9_ = 2.34	0.16	0.01	*F*_1,9_ = 0.74	0.41
	Stage and side	**0.03**	***F*_3,27_ = 3.4**	**0.03**	0.02	*F*_3,27_ = 1.88	0.15
*Interleaved* design	Stage	**0.23**	***F*_2,28_ = 5.74**	**0.008**	**0.22**	***F*_2,28_ = 5.74**	**0.008**
	Side	0.004	*F*_1,14_ = 0.92	0.35	**0.04**	***F*_1,14_ = 6.37**	**0.02**
	Stage and side	0.01	*F*_2,28_ = 1.3	0.28	**0.03**	***F*_2,28_ = 3.76**	**0.03**

*Significant interaction (bold) demonstrates a difference between the different stages that is dependent on the workspace-or-direction toward which the movement is applied.*

In the *interleaved* design, the analysis showed significant interaction between stage and direction (η^2^ = 0.03, *F*_2,28_ = 3.76, *p* = 0.03) (Figures 8C,D). We found significant elongation of leftward movements in both the end of Adaptation and at the beginning of the Washout (LA: *d* = 0.66, *t*_14_ = 2.62, *p* = 0.04, EW: *d* = 0.83, *t*_14_ = 3.05, *p* = 0.025). In addition, there was also a significant difference between leftward and rightward movements at the end of Adaptation (*d* = 0.65, *t*_14_ = 3.42, *p* = 0.004, Figure 8D). From these results we conclude that the dependency of the transfer effect of adaptation on workspace or direction is different between the two protocols of *blocked* and *interleaved*. The *interleaved* design is more sensitive in discovering transfer of adaptation, but nonetheless, we remain cautious in our answer to the second question about the dependency of the transfer of adaptation on workspace or direction.

Overall, from Experiment 1, we conclude that: (1) the adaptation to the asymmetrical delay in leftward movements *generalized to blind line bisection movements*, but not if they were rightward movements in the right workspace, and (2) we cannot determine whether the adaptation was workspace or direction dependent. From Experiment 2, we conclude that the generalization of the adaptation to the delay was *narrow and limited only to the lateral movements*.

### Perceptual Line Bisection Task

**FIGURE 9:**
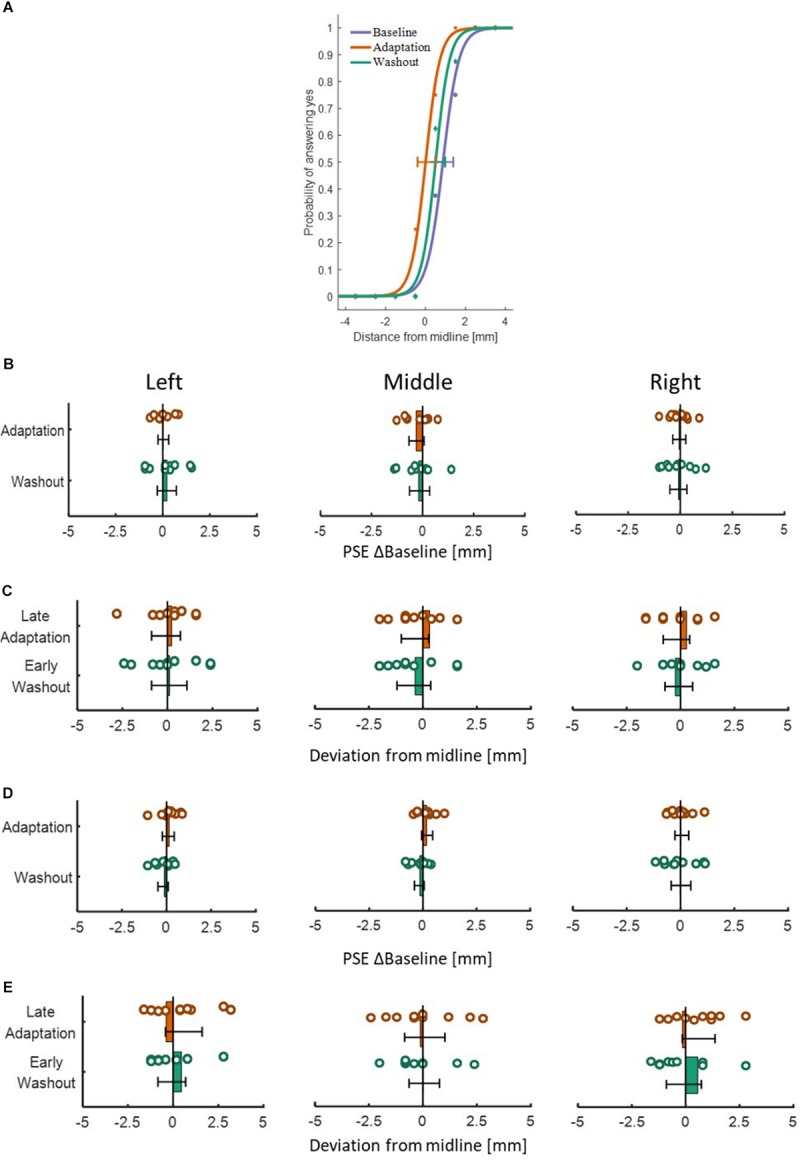
Results of the perceptual test in the different stages for both experiments and both protocols. **(A)** An example of three psychometric curves from a typical participant in the *blocked* design of Experiment 1. The dots represent the actual data from the different sessions of Baseline (purple), Adaptation (orange), and Washout (green), and solid lines are the fitted curves. Error bars represent 95% confidence interval. **(B)** PSE results for the *blocked* design in Experiment 1. Values are presented for the Adaptation (orange) and Washout (green) relative to Baseline. Colored circles represent the PSE value of each participant and error bars are 95% confidence interval. The bars are located spatially to represent the spatial direction of midline deviation. **(C)** Results of the perceptual test for the *interleaved* design in Experiment 1. We present the deviation in the end location of the probe compared to actual midline for Late Adaptation (orange) and Early Washout (green) relative to the end of Baseline. Circles and error bars are as in **(B)**. Panels **(D)** and **(E)** are same as **(B)** and **(C)** for the *blocked* and *interleaved* design in Experiment 2, respectively. Overall, no perceptual bias is demonstrated in both experiments and both protocols.

To examine the effect of asymmetrical delay on perception, in the *blocked* design we fitted a psychometric curve for each participant (examples are depicted in Figure 9A), and extracted the PSE value to determine the perceptual bias of the lines’ middle location. In the *interleaved* design, we extracted the difference between the end location of the probe and the actual midline. In both protocols, we used one-way repeated measures ANOVA model. The results showed that in both experiments and both protocols, the statistical analysis (as summarized in Tables 4, 5) yielded no significant effects on the perceived location of the midline between the different stages in the experiment (Figures 9B–E). These results clearly show that the perception was unaffected by adaptation to asymmetrical visuomotor delay.

**TABLE 4:** Results for the PSE value for each one of the presented lines in the two experiments.

	**Left**			**Middle**			**Right**		
	**η^2^**	***F*_2,18_**	***p***	**η^2^**	***F*_2,18_**	***p***	**η^2^**	***F*_2,18_**	***p***
Experiment 1	0.06	0.67	0.52	0.01	0.12	0.89	0.12	1.09	0.35
	Power = 0.99								
Experiment 2	0.13	1.44	0.26	0.26	3.18	0.06	0.002	0.02	0.97
	Power = 0.99								

*No significant effects were observed.*

**TABLE 5:** Results for the spatial deviation observed in the perceptual task of the *interleaved* design.

	**Left**			**Middle**			**Right**		
	**η^2^**	***F*_2,18_**	***p***	**η^2^**	***F*_2,18_**	***p***	**η^2^**	***F*_2,18_**	***p***
Experiment 1	0.006	0.05	0.94	0.13	1.31	0.29	0.04	0.41	0.06
	Power = 0.99								
Experiment 2	0.14	1.51	0.24	0.003	0.02	0.97	0.16	1.74	0.2
	Power = 0.99								

*No significant effects were observed.*

## Discussion

To understand the effect of laterality and time delays on action and perception, we investigated the effect of adaptation to laterally asymmetrical delay on movements and on visual perception. Following exposure to delay that was introduced exclusively in the left workspace, participants modified the extent of their reaching movements only in the left side, where the delay was applied. When participants were initially exposed to the delay, their leftward movements became hypermetric compared to the end of the Baseline session, i.e., they over reached the target. Throughout adaptation, they reduced the hypermetria, resulting in movements similar to those observed in the end of the Baseline. Additionally, aftereffects were observed when the delay was unexpectedly removed in terms of target undershoot. These results indicate that a workspace-specific internal representation was constructed to compensate for the movement errors caused by the perturbation.

In the transfer tasks, we observed that *the adaptation to the asymmetric delay only affected action, and not perception*. More specifically, we found that the effect of adaptation to a laterally asymmetrical delay transferred to the lateral blind motor line bisection task for left-workspace movements in one protocol, and to all leftward movements in another protocol. This effect was demonstrated in elongated movements compared to the movements performed before the exposure to the perturbation. Interestingly, only movements in the lateral direction were elongated, both leftward and rightward movements (Experiment 1), and movements that had substantial frontal component were not elongated (Experiment 2). In contrast, no effect was observed in the perceived midpoint of the presented lines in the *Perception* groups of both experiments in any of the protocols. Therefore, we conclude that the transfer of adaptation is dependent on the paradigm by which the participants were exposed to the perturbation, with a more pronounced and broader effect when the transfer trials were interleaved between the exposure trials.

### Adaptation and Representation of Visuomotor Delay

The effect of adaptation to a visuomotor delay on the execution of movements has been extensively investigated (Miall et al., 1985; Miall and Jackson, 2006; Honda et al., 2012; Rohde et al., 2014; Avraham et al., 2017a; Sulimani et al., 2017). Furthermore, the adaptation to delay that was presented only in one workspace was also examined (Avraham et al., 2018; Farshchian et al., 2018). In line with our results, Farshchian et al. (2018) found evidence for generalization of adaptation between left and right workspaces. However, our current results from the *interleaved* design are not consistent with our previous study with a similar adaptation to laterally asymmetric delay paradigm (Avraham et al., 2018), where transfer of adaptation was restricted to leftward movements in the left workspace. This might be because of the difference in the transfer task that was used in the two experiments. In our previous study we used circular drawing movements with multiple movement directions, whereas in the current study we used line bisection task with only lateral movements.

We found that the effect of adaptation to a left hemispace-specific delay during a reaching task transferred to the lateral (leftward and rightward) line bisection movements, but with a different manner according to the different protocols we tested. In the *blocked* design protocol, only movements that were performed in the left workspace were elongated, and only in the washout stage, while the *interleaved* design protocol yielded elongated leftward movements in both workspaces and during both LA and washout. In our previous study, we found that following adaptation to laterally asymmetric visuomotor delay in the left workspace, all the circles that were initiated in the left workspace were hypermetric (Avraham et al., 2018). By assuming a workspace-dependent generalization, we were able to explain the intriguing effect of adaptation to asymmetrical delay on transfer circular movements and to model a concept of perceptual-motor asymmetry in the hemispheres. However, in that experiment, workspace and direction were coupled, as all the movements started from the center. Here, our results showed that *laterally asymmetrical delay that was presented during leftward reaching movements has a pronounced transfer effect on blind leftward movements in the left-workspace and not on blind rightward movements in the right workspace*. There was also influence on leftward movements in the right workspace. In addition, even though we did not find a significant influence on rightward movements in the left workspace, the size of the mean change in hand amplitude was large. In light of the results from both *blocked* and *interleaved* design we conclude that the adaptation to asymmetrical delay transferred to leftward movements performed in the left workspace, and that the generalization to other directions or workspaces is dependent on the way participants were exposed to the perturbation and the exact protocol that was used for testing the transfer of adaptation.

Adaptation to asymmetrical delay transferred to the lateral blind line bisection movements. These movements can be considered as reaching movements toward the center of the presented line. Therefore, this result is in agreement with previous studies that showed hypermetric blind reaching movements after adaptation to delay (Botzer and Karniel, 2013; Avraham et al., 2018). These results are in agreement with the results of the *interleaved* design. However, in our *blocked* design protocol, the transferred effects were only observed in the Washout session, after the participants already practiced reaching movements without delay. This may indicate that the process of building an internal representation was slower in the blocked design than in the *interleaved* design. Consequently, even though no new information is being learned during the transfer blocks, they could have weakened the adaptation and cause a forgetting in the learning process as they interrupted the sequence of learning (Scheidt et al., 2000; Shmuelof et al., 2012). On the other hand, the *interleaved* design allowed for capturing the transfer of adaptation faster and highlighted that it generalized more broadly.

The way the sensorimotor system represents delay is still under dispute. On the one hand, studies have shown evidence for time-based representation (Witney et al., 1999; Levy et al., 2010; Rohde et al., 2014; Leib et al., 2015; Avraham et al., 2017b; Leib et al., 2018). On the other hand, other behavioral results demonstrated limited ability to represent time in the motor system, which raise the possibility for a state-based representation (Pressman et al., 2007; Sarlegna et al., 2010; Di Luca et al., 2011; Nisky et al., 2011; Takamuku and Gomi, 2015; Avraham et al., 2017a). Our results are consistent with a state-based representation, as the participants modified the extent of the reaching movements and exhibited aftereffects when the delay was removed. This implies that the participants did not represent the delay as a time-lag between the hand and the cursor.

### Visuomotor Adaptation and Perceptual Space Representation

We found no effect of motor adaptation on participants’ perceived midline, which shows that the space representation was unaffected by the adaptation process. This result is inconsistent with previous studies that showed transfer effects from action to perception (Ostry et al., 2010; Mattar et al., 2012; Marius‘t Hart and Henriques, 2016). However, in these studies perception was examined in terms of perceived direction and location of the hand, unlike in the current study in which we examined perception in terms of space representation. A similar result was also recently reported in a study that compared force field and prism adaptation by means of transferred effect to space representation (Michel et al., 2018). The results of this study showed no effect of force field adaptation on visual perception. In contrast, in the case of prism adaptation, transferred effects were observed in both control of action and space representation (Colent et al., 2000; Goedert et al., 2010; Fortis et al., 2018). Previous studies that compared delayed visual feedback and prism adaptation revealed different underlying mechanisms of adaptation between the two types of perturbations (Smith and Bowen, 1980). In addition, the observed difference can be related to the two learning processes theory (Smith et al., 2006); recent studies of prism adaptation suggested that the slow process is more dominant than the fast process (Michel et al., 2003), and that a third learning process is required in order to fully explain the decay of prism aftereffects after experiencing prism adaptation for 500 trials (Inoue et al., 2014). These characteristics of prism adaptation might be the cause for the different transfer of perceptual effects in comparison with our results and the results of force field adaptation. Another reason for potential discrepancy may be the stronger realism of adaptation to prism goggles compared to the virtual reality scenario in our setup.

There is an ongoing controversy about the existence of two distinct pathways for action and perception in the visual system. One view suggests that there are two separate pathways for processing of visual information for perception and for control of action (Goodale and Milner, 1992). This idea is supported by behavioral evidence for independent processing of information for perception and action in grasping (Aglioti et al., 1995; Ganel and Goodale, 2003; Milstein et al., 2018), and lifting (Flanagan and Beltzner, 2000). Alternatively, evidence suggested that action and perception might be intertwined in some cases (Franz et al., 2000; Smeets and Brenner, 2006; Reichenbach and Diedrichsen, 2015). According to this view, the observed dissociation between action and perception could be a result of different types of measures and environmental cues that are affecting each of the processes differently (Smeets and Brenner, 2006). However, this entire line of research did not examine motor adaptation effects, except from very fast adaptation of grip force during lifting (Flanagan and Beltzner, 2000). Here, we showed that when breaking the simultaneity between the visual and proprioceptive input, participants’ perceptual space representation remained unaffected. Therefore, when participants were asked to report the perceived location of a presented midline, no deviation was observed. However, their lateral movements toward the midline are modified when no visual feedback is provided. While our results demonstrate a dissociation between processing of visual information for action and perception following adaptation to visuomotor delay, we interpret them in the context of motor adaptation processes that affect differently transfer to action and perception (Ostry et al., 2010; Mattar et al., 2012; Marius‘t Hart and Henriques, 2016), rather than in the context of the different pathways in processing of visual information (Goodale and Milner, 1992; Milner and Goodale, 2006). Future studies are needed to examine potential interconnections between these two separate lines of research.

Our results showed transfer effects to the control of action but not to perceptual space representation. In the *blocked* design, this difference between action and perception could have stemmed from the large amount of data that are required for generating a psychometrical curve, which might have affected the learning sequence. However, in the *interleaved* design, the perceptual task did not require such large amount of data, and was very similar to the action task, excluding the planning and execution of a reaching movement. Therefore, we conclude that the results of the perceptual task from the *interleaved* design are more appropriate for comparison with the action task than the block design. Nevertheless, the conclusions of both protocols were consistent showing that there was no transfer of adaptation to a bias in perception.

In our previous work, we found spatial deviations after adaptation to laterally visuomotor delay. We explained these results with a model for perceptual and motor asymmetry in the hemispheres. However, it is noteworthy to distinguish between the unbiased perception discussed in the current study and the perceptual dominance component of the model in our previous study (Avraham et al., 2018). In the present study, perception is interpreted as the spatial representation that is reported by the subject. In contrast, in our previous study, perception is referred to the space representation in the hemispheres which forms our motor behavior across space. Accordingly, the observed dissociation between action and perception does not contradict our proposed model for perceptual and motor asymmetry in the hemispheres that explain the motor effects.

### Hemi-Spatial Neglect and Hyperschematia

Neglect patients fail to perceive and respond to stimuli presented on the side contralateral to their lesion. Studies on neglect patients showed that the foundation of neglect is a deficit in both perceptual space representation and motor behavior across space (Marotta et al., 2003; Adair and Barrett, 2008; Rossit et al., 2012). The motor impairments can be demonstrated in temporal disorders of slowness in movement initiation (directional hypokinesia) or in execution of movements (directional bradykinesia), and unilateral spatial disorders of reduction in movement amplitude (directional hypometria) (Mattingley et al., 1992, 1994). Moreover, the motor impairment can also be observed in leftward movements performed in the right workspace (Danckert and Ferber, 2006). In the current study, our motor task yielded neglect-like elongated line bisection movements. However, our perceptual line bisection test results showed no midline perceptual biases. Therefore, we conclude that temporal processes cannot be addressed as the main neural basis of neglect, but they might be associated with the spatial motor distortions in neglect, and can be used as a rehabilitation technique in cases of severe motor impairment.

Another pathology is the “hyperschematia,” in which patients exhibit leftward enlargement of drawings both when copying an object or drawing from memory (Rode et al., 2014). This disorder is more frequent after right-brain damage, and the patients are unaware to their deficit (Rode et al., 2018). In the current study, left-workspace lateral movements were elongated after exposure to laterally asymmetrical delay, and no effect on perception was observed. Therefore, we suggest that the disorder in hyperschematia might be related to visuo-temporal processing. However, further investigation is required.

Understanding the functional lateralization in the hemispheres and related behaviors when presented with temporal and spatial perturbations may help us to better understand pathological cases involving injury in only one hemisphere manifesting in misperception of the environment as well as motoric impairments. By deepening our understanding, we might be able to develop new and improved diagnostic and rehabilitation methods to help patients with these complex syndromes.

## Ethics Statement

This study was carried out in accordance with the recommendations of the Human Subjects Research Committee of Ben-Gurion University of the Negev, Beersheba, Israel with written informed consent from all subjects. All subjects gave written informed consent in accordance with the Declaration of Helsinki. The protocol was approved by the Human Subjects Research Committee of Ben-Gurion University of the Negev, Beersheba, Israel.

## Author Contributions

CA, MD, HK, GA, FM-I, and IN designed the experimental protocol and hypotheses, interpreted the results, edited the manuscript, and approved the final version of the manuscript. MD and HK performed the experiments. CA, MD, and HK analyzed the data and wrote the first draft of the manuscript.

## Conflict of Interest Statement

The authors declare that the research was conducted in the absence of any commercial or financial relationships that could be construed as a potential conflict of interest.
